# Revisiting the Relationship Between the Strength of Environmental Regulation and Foreign Direct Investment

**DOI:** 10.3389/fpsyg.2022.899918

**Published:** 2022-05-12

**Authors:** Moon Gyu Bae, Yi Chen Wang, Na Liu

**Affiliations:** ^1^Institute of Management and Economy Research, Yeungnam University, Gyeongsan, South Korea; ^2^School of Business, Guangdong Polytechnic of Science and Technology, Guangzhou, China; ^3^Department of International Business and Economics, Yeungnam University, Gyeongsan, South Korea

**Keywords:** FDI, environmental regulation, country distance, ESG, pollution haven hypothesis, porter hypothesis, sustainability

## Abstract

Interest in sustainability is increasing, and research on ESG management continues. The first issue to be discussed in the present situation is the environment. The study between the environment and internationalization was conducted around two conflicting arguments. First, the pollution haven hypothesis states that multinational corporations move to countries with looser regulations depending on environmental regulation. Next is the Porter Hypothesis, which argues that well-designed environmental regulations offset the cost of compliance and ultimately help firms gain a competitive advantage through innovation that enhances performance. However, the two arguments have not yet reached a consensus conclusion. In addition, studies on the national level and studies considering the distance between countries, an important factor in international management, are lacking. This manuscript aims to revisit the relationship between the strength of environmental regulation and foreign direct investment (FDI) in the context of increasing environmental concerns. Differences between countries are an important field of international management, but research on environmental regulations is lacking. The purpose of this study is to examine the relationship between existing environmental regulations and FDI and to discuss how the distance between countries can affect existing theories.

## Introduction

An important motive for firms has always been to generate profits and maximize shareholder value. However, as the environmental and social issues emerge, the focus of governments, institutions, investors, and firms is shifting toward more ‘socially responsible’ behaviors of firms along with maximizing profits. This concern has garnered much attention as a way of assessing the non-financial performance of companies, such as environmental, social, and governance (ESG) issues and ethical considerations. Currently, ESG management has become an important issue. According to Morgan (2020), economic policymakers and investment decision-makers should make a wake-up call to sustainability management. The US SIF Foundation Report found that portfolio investors consider ESG factors at a 42% higher rate than in 2018, and ESG investments are one for all assets invested in the United States. 1/3 (US SIF, 2020).

The pressure on sustainability management, which started with CSR, that firms are receiving continues. The pressure on the firm is divided into moral and strategic obligations. Moral obligations have a role in resolving social problems because a company has obligations to shareholders and external stakeholders (Freeman, 1984; Carroll, 1991; Wood, 1991), and strategic obligations can improve corporate competitiveness through sustainable management (Porter and Van der Linde, 1995; Russo and Fouts, 1997; Porter and Kramer, 2011). Studies dealing with these two obligations have received significant attention at the firm level, but studies at the country level are still lacking. Considering the growing interest in ESG worldwide and the agreement on the Sustainable Development Goals (SDGs), national-level research is necessary.

In an increasing interest in ESG management, the priority area to be discussed is the environment. Concerns about the future have led to actions demanding a better environment (Sharfman et al., 2004). The greatest challenge of the 21st century is balancing environmental degradation with economic growth (Alola, 2019). Countries made efforts to achieve the two goals of high-quality economic development and an eco-friendly ecosystem, and foreign direct investment (FDI) was emphasized to achieve the goals. Research on FDI and the environment was conducted centered around the study of the effect of the strength of environmental regulations and two theories located at both extremes. The pollution haven hypothesis (PHH) explains that countries make environmental regulations looser to attract FDI. Depending on the degree of environmental regulation, multinational corporations with pollution-intensive industries move to countries with looser regulations. Contrary to the PHH theory, the Porter Hypothesis (1991) suggests that well-designed environmental regulations offset the cost of compliance, leading to innovations that improve corporate performance (Porter and Van der Linde, 1995). It is explained that it mediates the development and adoption of green innovation to help secure a competitive advantage that affects corporate performance (Xing et al., 2019).

However, many existing studies dealing with the two theories have been discussed at the corporate level. To the best of our knowledge, few studies have addressed the inter-country distance between investment and host countries dealing with environmental regulation and FDI (Bu and Wagner, 2016). “Distance” has been used as a metaphor for differences between countries in international management (Shenkar, 2012). Research in international management mentions “distance” as a major factor impacting foreign direct investments and location choices (Beckerman, 1956; Johanson and Wiedersheim-Paul, 1975; Zaheer, 1995; Shenkar, 2001; Blomkvist and Drogendijk, 2013). Several recent IB (International Business) studies have been conducted on the relationship between country distance and FDI (Evans et al., 2000; Sousa and Bradley, 2006; Brewewer, 2007; Child et al., 2009). In other words, the purpose of this study is to examine the relationship between existing environmental regulations and FDI and to discuss how country distance can affect existing theories.

## Background and Literature Review

The debate over environmental regulations and FDI attraction has been the focus of various theories (Santos and Forte, 2021). The first is that multinationals tend to invest in countries with less stringent environmental regulations because cost considerations are important when choosing a host country. This phenomenon is called the Pollution Haven Hypothesis (PHH). Therefore, they are easily attracted to developing countries where environmental regulations are perceived to be less stringent in investment location decisions (Cole et al., 2017). The second theory is the pollution halo effect. For example, if a technologically advanced multinational corporation decides to invest in an area that is less technologically advanced, it is positive if the latter helps reduce pollution, but if the company decides to move to a less regulated area and the pollution in that area is negative if it contributes to an increase in level (Cole et al., 2017). A third theory could lead to an effect known as “race to the bottom” (RTB) in which host countries seek to ease local environmental regulations to attract FDI (Cole et al., 2017). The fourth theory is the environmental Kuznets curve (EKC), which hypothesizes the relationship between economic development and the environment. The impact of FDI on the promotion of industrialization will be examined. The main content is that incomes will also increase because of economic development and wealthier population groups will demand agencies to enforce environmental regulations and ensure better quality for the environment, thus leading to reduced pollution (Zugravu-Soilita, 2017). Finally, a fifth theory is the Porter hypothesis-based mechanism in environmental regulation theory is that well-designed environmental regulations stimulate innovation that offsets regulatory compliance costs and ultimately improves firm financial performance (Porter and Van der Linde, 1995).

Although there is such extensive literature on the subject, RTB is an extension of the PHH theory, and environmental regulation is not central to the pollution halo effect. EKC focuses on the dynamics that change according to the time and the condition of the host country. In conclusion, the theories that the host country’s environmental regulation focuses on are the PHH and the Porter hypothesis. This study was conducted with a focus on literature research on PHH and the Porter hypothesis.

Psychological research tends to favor familiar situations over unfamiliar situations (Powell and Ansic, 1997). International management conducted a study on the concept of distance between countries that implicitly captures these insights. And the importance of the street is represented by the sentence “International management is the management of the distance” (Zaheer et al., 2012). This article suggests that differences between home countries and host countries can create uncertainties between countries and influence their decisions in the internationalization process (Child et al., 2009). The distance between countries was defined and used in various concepts. In this study, the distance between countries is focused on the four characteristics proposed by Ghemawat (2001) and enables a comprehensive view of the relationship between environmental regulations and FDI.

## Pollution Haven Hypothesis and Porter’s Hypothesis

The pollution haven hypothesis (PHH) has been debated for decades between internationalization and environmental pollution. It is argued that trade and capital movement liberalization contribute to the transfer of polluting industries from countries with relatively strict environmental regulations to countries with less stringent regulations (Hille, 2018). For this reason, stringent regulation of environmental standards leads to new equipment requirements, landfill rules, restrictions on specific inputs and outputs, and additional production costs due to the need to find alternative methods for waste disposal. This is because investment is shifted to countries with relatively less stringent regulations (Rezza, 2015). PHH focuses on the cost-effectiveness of environmental regulations considered by enterprises. The difference in production costs is a sufficient stimulus for enterprises to relocate to production facilities. Assuming that increased production costs are sufficient reasons for firms to move, firms are usually associated with replacing certain production lines, using different equipment, or finding new methods. The primary argument of PHH is that pollution is a factor of production, and in countries with low pollution costs, producers should use the pollutant intensively (Bu and Wagner, 2016). In conclusion, PHH is a representative theory showing a negative relationship between environmental regulation and FDI, and PHH becomes a leading theory of race to the bottom.

In the face of a race to the bottom from countries with weak environmental regulations, the conditions under which firms decide to attract investment respond to differences in environmental regulations (Erdogan, 2014). Previous studies assume that all firms in the industry are equally affected by the structures of environmental regulations, and the host country’s government is under tremendous pressure to be environmentally flexible (Madsen, 2009). When attracting FDI, the host country takes a strategic position through legislation and faces a dilemma. Two situations can be assumed. First, choosing public welfare as a priority will compete with a race to the top. Still, the inflow of investment in the polluting industry decreases (Erdogan, 2014), and the withdrawal of investments can hurt the national economy. In the opposite case, if the decision to attract FDI by reducing environmental regulations related to the polluting industry, multinational companies will be relocated to looser-regulated countries and accept long-term environmental destruction in exchange for short-term economic benefits while inducing a transition (Madsen, 2009).

Regarding the relationship between environmental regulation and internationalization, many studies examined, at the environmental level, the contents of environmental regulation policies and their effect on globalization *via* internationalization. Country-level (Kahouli and Omri, 2017; Mulatu, 2017; Zugravu-Soilita, 2017), industry-level (Cole and Elliott, 2005), and firm-level research (Javorcik and Wei, 2003; Dam and Scholtens, 2012) were variously studied. A study covering China’s economic revival and environmental pollution relationship went ahead (Bu et al., 2013; Cai et al., 2018; Cheng et al., 2018).

Various empirical studies have been conducted to confirm PHH in internationalization. Mulatu (2017) checked the PHH through the relationship between environmental regulations and FDI in 64 investment-inducing countries for 4 years to investigate the possibility of UK-based multinational companies entering a country with loose environmental regulations. It was confirmed that multinational companies belonging to polluting industries tend to invest in countries with loose environmental regulations. Naughton (2014) analyzed the effect of home country environmental regulation on FDI, deviating from the focus of host country environmental regulation intensity, and concluded a correlation between FDI and strict environmental regulation.

Studies to identify PHH show mixed results (Cole and Elliott, 2005; Dietzenbacher and Mukhopadhyay, 2007; Tang, 2015; Zugravu-Soilita, 2017). Dietzenbacher and Mukhopadhyay (2007) showed that PHH is the result of the Heckscher-Ohlin theory through input-output analysis, and in India, the opposite was shown. However, depending on the characteristics of imported products, the PHH theory was contradictory (Cave and Blomquist, 2008).

Economic evidence from empirical studies have suggested that environmental regulation and financial performance are closely linked (Ambec et al., 2013). However, it remains controversial whether environmental regulations are an efficient mechanism to achieve sustainability and improve financial performance (López-Gamero et al., 2010; Testa et al., 2011). PHH has been recognized as a negative mechanism for firm performance and the environment regarding environmental regulations and firm performance. However, PHH shows contradictory results. Contrary to PHH theory, the Porter hypothesis-based mechanism in environmental regulation theory is that well-designed environmental regulations stimulate innovation that offsets regulatory compliance costs and ultimately improves firm financial performance (Porter and Van der Linde, 1995). To this end, the government designs and implements the “right type” of policies. Shifting environmental concerns into a competitive advantage requires establishing the correct type of regulation, which leads to processes that reduce pollution and costs or improve quality (Porter, 1991). In particular, the ‘right type of regulation’ is a tool that leads to new technological solutions and innovations, which in turn improves the allocation of resources.

Previous studies related to the Porter hypothesis have studied the positive relationship between corporate innovation and environmental regulation. Companies responded to the increasing intensity of environmental regulations through innovation, showing a positive relationship between environmental regulations and innovation (Lanjouw and Mody, 1996; Popp, 2006). Frondel et al. (2007) used OECD data and observed that strict environmental regulations tend to positively affect cost savings, general management systems, and specific environmental management tools have a positive effect on clean production. Horbach et al. (2013) argued the importance of regulation as a driver of eco-innovation compared to other innovations in German and French firms in a two-country comparison. Also, studies related to the Porter hypothesis were conducted on the relationship between internationalization performance and environmental regulations. Among polluting industries, specific industries are determined by the host country’s loose environmental regulations, but do not affect all industries (Xing and Kolstad, 2002).

Furthermore, countries with environmental stringency below a certain level are less attractive for investment (Kalamova and Johnstone, 2012). As environmental regulations become more assertive, it does not necessarily show negative consequences for FDI inflow (Waldkirch and Gopinath, 2008; Costantini and Mazzanti, 2012). Waldkirch and Gopinath (2008) found that less pollution-intensive industries invest more FDI in Mexico, suggesting that environmental regulations that enforce emission reductions may not necessarily harm FDI inflows. Costantini and Mazzanti (2012) showed that energy tax positively affects high-tech and low-medium-tech exports of 14 EU exporting countries and that environmental policies induce innovative performance mechanisms. Furthermore, environmental regulations can strengthen a country’s comparative advantage in exports (Costantini and Crespi, 2008; Groba, 2014).

FDI can promote both races to the bottom and the top (Madsen, 2009). In the former case, firms operating in a specific country will increase their costs due to increased environmental regulations. Firms will import or relocate production plants or pollution-intensive products to foreign countries with less stringent regulations (Jaffe et al., 1995). In other words, loose environmental regulations can be a location advantage. Global competitive pressures can motivate multinationals to choose countries with relatively loose environmental regulations (Dasgupta et al., 2002). As a result, foreign investment decisions are expected to be made in countries with lax environmental standards (Kalamova and Johnstone, 2012). In the latter case, environmental regulation induces innovation, which positively affects productivity and increases profitability (Porter and Van der Linde, 1995). Multinational corporations can have high clean technology and high-quality environmental management systems due to stricter environmental regulations than the host country (Zarsky, 1999). The social and environmental responsibilities that multinational corporations receive lead to demands for corporate strategy. As a result, multinational companies in developed countries are expected to make more significant green investments than companies in developing countries, and eco-innovation is likely to occur larger than in SMEs (Blomström and Kokko, 1998; Eskeland and Harrison, 2003). Therefore, it is very likely that MNEs will develop specific environmental technology and management standards and then apply them to foreign facilities, making it relatively easy to transfer knowledge from home to foreign countries and vice versa (Dasgupta et al., 2002). In conclusion, FDI transfers innovation from an investment country to a host country and ultimately enables additional innovation (Lanjouw and Mody, 1996).

## Country Distance

According to Hymer (1960), the liability of foreignness is likely to prevail over foreign firms because domestic firms have the general advantage of obtaining better information about their countries, such as economy, language, law, and politics. In addition, the cost of obtaining information for foreign firms may be high, and it is a barrier to the international operation cause of discrimination by the government, consumers, and suppliers. Liability of foreignness refers to additional costs incurred when a firm operates a business abroad. Specifically, they consist of spatial distance, inexperience in the host countries’ environment, discrimination in investment host countries, and expenses imposed by the host countries’ environment (Zaheer, 1995).

The liability of foreignness stems from differences between countries. And differences between countries were used as a metaphor for “distance” in international management (Shenkar, 2012). Country distance is one of the most widely studied and controversial concepts related to distance in international management and marketing (Shenkar, 2001). In general, distance can be measured by an individual, a team, an organization, a country, a language group, an ethnic group, or the distance between two entities. Distance has a metaphorical meaning that refers to group differences between countries beyond simply geographic and physical (Zaheer et al., 2012). Commonalities between countries (signs of similarity) close the distance, and differences (signs of dissimilarity) make countries farther apart. That is, the commonalities of the home country and the potential host country favor entry (Williams and Grégoire, 2015).

Country distance has been described in various ways. First, psychic distance is the sum of factors that impede the flow of information between firms and markets (Johanson and Wiedersheim-Paul, 1975). Cultural differences and uncertainties degree caused by various factors that hinder the learning and operation of overseas markets (O’Grady and Lane, 1996). Since then, psychic distance has been extended to various factors that make the distance longer. Various factors identified four dimensions of Cultural, Administrative, Geographic, and Economic (CAGE) (Ghemawat, 2001), the dominant religion, business language, form of government, economic development (Boyacigiller, 1990), language, business practice, political and legal systems, education, economic development, marketing, infrastructure, and industrial structures (Evans et al., 2000).

Although FDI suggests that FDI is the preferred entry mode because it allows firms to transfer knowledge and other assets without relinquishing ownership or management, it can lead to problems and conflicts related to the liability of foreignness (Johanson and Vahlne, 2009; Berry et al., 2010). Country distances have been highlighted in various fields, including FDI-related internationalization outcomes, entry methods, market selection, internationalization processes, antecedents, determinants, coping measures, and activities in other areas (Ciszewska-Mlinarič and Trąpczyński, 2016). Blomkvist and Drogendijk (2013) found that country distance is influenced by some psychic distance stimuli, including the integrative composition of distance and differences in culture, religion, democracy, and language. Distant country distances increase the cost of tailoring goods and services to local tastes and preferences (Miller and Eden, 2006) and the difficulty in overcoming discrimination and litigation (Hennart et al., 2002; Mezias, 2002). It has much more significant difficulties in establishing and maintaining business relationships in the host country (Slangen et al., 2011), which can negatively affect performance and influence overseas expansion decisions (Hennart et al., 2002; Flores and Aguilera, 2007). As the distance increases, it is more difficult for MNCs to acquire market knowledge, making them less competitive than the host country (Zaheer, 1995).

In this study, the distance between countries is focused on the four characteristics proposed by Ghemawat, 2001:cultural, administrative, geographic, and economic distance. First, culture is one of the most frequently cited and empirically tested factors contributing to country distance (Sousa and Bradley, 2006). Cultural distance refers to a cultural country that creates uncertainty and increases costs by limiting the flow of information and knowledge between countries and finding a negative relationship between entry into foreign markets (Berry et al., 2010).

Second, various government policies are an essential source of administrative distance (Ghemawat, 2001). The presence of corruption in the target country, a high barrier to foreign market entry (Weitzel and Berns, 2006), leads to an increase in administrative distance between countries. Countries with weak institutional systems and corruption are more likely to prefer FDI from countries with close administrative distance (Hotchkiss, 1998). Since administrative distance incurs high coordination costs, it is reasonable to assume that administrative distance will increase barriers to entry. Various studies have shown administrative factors such as language (Johanson and Vahlne, 1977), religion (Ghemawat, 2001), or legal system (Berry et al., 2010) have a strong influence on corporate strategic decisions and emphasize differences in political (Henisz, 2000) and trade relationships (Fratianni and Oh, 2009). Most studies on the effect of administrative distance on foreign market entry show a negative relationship (Berry et al., 2010; Guler and Guillén, 2010).

Third, various studies in the field of international management frequently use geographic distance to study the international activities of firms (Bevan and Estrin, 2004). Unlike the previous abstract concept, the geographic distance as a physical concept generally suffers from difficulties in business operation as the distance between two countries increases. Since it is associated with an increase in transportation and communication costs, the cost of dispatching foreign workers, and the costs associated with overcoming cultural, linguistic, and regulatory differences (Ghemawat, 2001; Berry et al., 2010).

Finally, a country’s economic development has traditionally been viewed as a reflection of the country’s market potential (Evans and Mavondo, 2002). However, Mitra and Golder (2002) found that the extensive economic distance between the home country and the host country prevented entry into foreign markets because consumers in countries with similar per capita GDP had similar consumption patterns and similar marketing strategies. In previous studies, the concept of economic distance represents important factors such as differences in customer preferences, differences in purchasing power, and differences in transportation and communication infrastructure. Firms are more likely to succeed by entering a country with an economic environment like their home country because, first, firms can more easily transfer their existing business models to countries with economic characteristics like their home country (Malhotra et al., 2009). Second, by entering a country that is economically like the home market, a firm can build an economy of scale, scope, and experience through the transfer of technology and knowledge from the home market to the host country’s market. International experience can also be enhanced by operating in similar countries and expanding to more economically distant countries (Malhotra et al., 2009).

## Discussion

### Summary

When globalization is accelerating, there is constant debate about sustainability management research. Among them, the environment is one of the pillars of ESG management and it has become an important issue. The pressure on sustainability management, which started with CSR, that firms are receiving continues. The pressure on the firm is divided into moral and strategic obligations. Moral obligations have a role in resolving social problems because a company has obligations to shareholders and external stakeholders (Carroll, 1991; Freeman, 1984; Wood, 1991), and strategic obligations can improve corporate competitiveness through sustainable management (Porter and Van der Linde, 1995; Russo and Fouts, 1997; Porter and Kramer, 2011). Studies dealing with these two obligations have received significant attention at the firm level, but studies at the country level are still lacking. Considering the growing interest in ESG worldwide and the agreement on the Sustainable Development Goals (SDGs), national-level research is necessary. Studies dealing with the relationship between environmental regulations and globalization performance in the environmental aspect have been approached in several ways, including various countries, entry methods, and corporate performance, but an agreed conclusion is still lacking. To the best of our knowledge, few studies have addressed the inter-country distance between investment and host countries in studies dealing with environmental regulation and FDI (Bu and Wagner, 2016). In other words, the purpose of this study is to examine the relationship between existing environmental regulations and FDI and to discuss how country distance can affect existing theories.

We propose the importance of country distance in the study of the relationship between environmental regulation and FDI. The reason is that PHH assumes that strong regulation leads to higher costs. However, starting with the Kyoto Protocol in 1997, the types and power of various pan-national regulations such as the Paris Agreement in 2015 are increasing. If the regulations are the same, the aspect that needs to be carefully considered in the entry conditions of countries is the distance between countries, which needs to be re-examined. The next important assumption in the Porter hypothesis is ‘well-designed regulation.’ The distance between countries has the potential as a proxy for a well-designed and regulated variable in the host country. A well-designed regulation is that if you are considering the host country with high environmental regulations, your investment decision will depend on how far/close that country is. The increased commonalities discussed earlier makes it easier to adapt to regulations. In other words, a well-designed regulatory perspective can be thought of as the relative effect of distances between countries.

This manuscript suggested examining the effect of current environmental regulations, which concentrate on the environment, on FDI, and the effect of the relationship between environmental regulations and the country distance on FDI.

### Environmental Regulation as National Competitiveness

Competitiveness equates to the ability to achieve specific outcomes, such as high living standards and economic growth, or focuses on achieving specific economic outcomes, such as job creation, exports or FDI, low wages, and stable unit labor costs. It is defined in several ways, including specific regional conditions, such as a “competitive” exchange rate to support a balanced budget or current account surplus (Delgado et al., 2012). For example, countries like Sweden show a high quality of life, and countries like China show competitiveness in driving growth through low labor costs. A new aspect of competitiveness is innovation. For example, improved products and processes arising from regulatory R&D and innovation (Testa et al., 2011), corporate reputation, and green credentials are also competitive (Poelhekke and Van der Ploeg, 2015). While previous studies have found that environmental regulation promotes innovation, the most significant conflict is the effect of environmental regulation on competitiveness (Cohen and Tubb, 2018). Early research found that environmental regulation harmed productivity (Palmer et al., 1995), but it might be reasonably assumed that environmental regulation had a positive effect on productivity (Berman and Bui, 2001; Lanoie et al., 2008).

From the perspective of national competitiveness, if environmental regulations are extensive in terms of cost, environmental competitiveness is low. However, according to Porter’s hypothesis, environmental competitiveness is high. This paper shows that strict environmental regulations might have a positive relationship with FDI is contrary to the cost perspective of previous studies. Conflicting results require discussion of other aspects of environmental regulation. In addition to implementing innovation to comply with environmental regulations, countries with strict environmental regulations are likely to have high environmental technology levels, and companies with high environmental technology are likely to have standards that are difficult to match with other companies, duplication, and application of conformity assessment procedures. There is a possibility of various restrictions such as transparency in-laws and technical regulations. Companies will enter countries with high environmental regulations to take a preemptive response. Strict environmental regulations provide an opportunity to raise an ethical reputation, which can be a new asset. This paper proposes the possibility of environmental regulation as a factor of national competitiveness through previous discussions.

## Author Contributions

MB and NL conceived of the presented idea. MB wrote the first draft of the manuscript. YW critically revised the manuscript. All authors discussed the results and contributed to the final manuscript, and approved the final version of the manuscript.

## Conflict of Interest

The authors declare that the research was conducted in the absence of any commercial or financial relationships that could be construed as a potential conflict of interest.

## Publisher’s Note

All claims expressed in this article are solely those of the authors and do not necessarily represent those of their affiliated organizations, or those of the publisher, the editors and the reviewers. Any product that may be evaluated in this article, or claim that may be made by its manufacturer, is not guaranteed or endorsed by the publisher.
